# Long‐lasting correction of in vivo LTP and cognitive deficits of mice modelling Down syndrome with an α5‐selective GABA_A_ inverse agonist

**DOI:** 10.1111/bph.14903

**Published:** 2020-01-09

**Authors:** Arnaud Duchon, Agnès Gruart, Christelle Albac, Benoît Delatour, Javier Zorrilla de San Martin, José María Delgado‐García, Yann Hérault, Marie‐Claude Potier

**Affiliations:** ^1^ Translational Medicine and Neurogenetics Institut de Génétique et de Biologie Moléculaire et Cellulaire Illkirch France; ^2^ Centre National de la Recherche Scientifique UMR7104 Illkirch France; ^3^ Institut National de la Santé et de la Recherche Médicale U1258 Illkirch France; ^4^ Neuropôle Université de Strasbourg Illkirch France; ^5^ División de Neurociencias Universidad Pablo de Olavide Seville Spain; ^6^ Institut du Cerveau et de la Moelle épinière Hôpital de la Pitié‐Salpêtrière Paris France; ^7^ Institut National de la Santé et de la Recherche Médicale U1127, Hôpital de la Pitié‐Salpêtrière Paris France; ^8^ Centre National de la Recherche Scientifique UMR7225, Hôpital de la Pitié‐Salpêtrière Paris France; ^9^ Sorbonne Université Hôpital de la Pitié‐Salpêtrière Paris France

## Abstract

**Background and Purpose:**

Excessive GABAergic inhibition contributes to cognitive dysfunctions in Down syndrome (DS). Selective negative allosteric modulators (NAMs) of α5‐containing GABA_A_ receptors such as the α5 inverse agonist (α5IA) restore learning and memory deficits in Ts65Dn mice, a model of DS. In this study we have assessed the long‐lasting effects of α5IA on in vivo LTP and behaviour in Ts65Dn mice.

**Experimental Approach:**

We made in vivo LTP recordings for six consecutive days in freely moving Ts65Dn mice and their wild‐type littermates, treated with vehicle or α5IA. In parallel, Ts65Dn mice were assessed by various learning and memory tests (Y maze, Morris water maze, or the novel object recognition) for up to 7 days, following one single injection of α5IA or vehicle.

**Key Results:**

LTP was not evoked in vivo in Ts65Dn mice at hippocampal CA3‐CA1 synapses. However, this deficit was sustainably reversed for at least six consecutive days following a single injection of α5IA. This long‐lasting effect of α5IA was also observed when assessing working and long‐term memory deficits in Ts65Dn mice.

**Conclusion and Implications:**

We show for the first time in vivo LTP deficits in Ts65Dn mice. These deficits were restored for at least 6 days following acute treatment with α5IA and might be the substrate for the long‐lasting pharmacological effects of α5IA on spatial working and long‐term recognition and spatial memory tasks. Our results demonstrate the relevance of negative allosteric modulators of α5‐containing GABA_A_ receptors to the treatment of cognitive deficits associated with DS.

**What is already known**

- The α5 inverse agonist (α5IA) reverses cognitive deficits in Ts65Dn mice modelling Down syndrome.

**What does this study add**

- α5IA produces long‐lasting pharmacological effects by sustainably restoring in vivo LTP in Ts65Dn mice.

**What is the clinical significance**

- These results highlight the interest of α5IA for treating cognitive deficits in Down syndrome.

**Abbreviations**
α5IA: 1‐methyl‐4‐({[3‐(5‐methyl‐1,2‐oxazol‐3‐yl)‐[1,2,4]triazolo[3,4‐a]phthalazin‐6‐yl]oxy}methyl)‐1H‐1,2,3‐triazole (α5 inverse agonist)
DS: Down syndrome
fEPSP: field EPSP
HFS: high frequency stimulation
IEG: immediate early gene
MWM: Morris water maze
NAM: negative allosteric modulator
NOR: novel object recognition

## 1 INTRODUCTION

Down syndrome (DS) is the consequence of a third complete or partial copy of human chromosome 21 (Antonarakis, 2017; Antonarakis, Lyle, Dermitzakis, Reymond, & Deutsch, 2004; Lejeune, Gautier, & Turpin, 1959). It is associated with physical abnormalities including characteristic facial features and cognitive deficits producing variable impairments in intellectual functioning. In recent years, beside cognitive therapies that have proven to be useful for improving deficits in DS, active research has identified possible pharmacological treatments in mouse models (Martinez‐Cue, Delatour, & Potier, 2014; Ruparelia, Pearn, & Mobley, 2012; Stagni, Giacomini, Guidi, Ciani, & Bartesaghi, 2015). Several mouse models have been extremely useful for deciphering the mechanisms involved in cognitive defects in DS and for the design of preclinical studies (Herault et al., 2017; Sheppard, Wiseman, Ruparelia, Tybulewicz, & Fisher, 2012). The most widely used mouse model is the Ts(17^16^)65Dn, Ts65Dn mouse (Reeves et al., 1995). These mice, carrying a partial *Mmu*16 trisomy for 122 genes, reproduce DS memory defects and show deficits in LTP on hippocampal slices, that are reversed by non‐selective antagonists of https://www.guidetopharmacology.org/GRAC/FamilyDisplayForward?familyId=72 (Kleschevnikov et al., 2004). In addition, GABA_A_ antagonists restore cognitive impairments in Ts65Dn mice (Colas et al., 2013; Colas, Chuluun, Garner, & Heller, 2017; Fernandez et al., 2007; Rueda, Florez, & Cue, 2008).

Previously, we demonstrated that a single dose of the α5 inverse agonist (https://www.guidetopharmacology.org/GRAC/LigandDisplayForward?ligandId=4095), a selective negative allosteric modulator (NAM) of https://www.guidetopharmacology.org/GRAC/ObjectDisplayForward?objectId=408_A_ receptors containing the α5 subunit (Dawson et al., 2006), was able to rescue learning and memory deficits and to promote immediate early gene (IEG) synthesis in the hippocampus of Ts65Dn mice (Braudeau et al., 2011; Braudeau et al., 2011). Subsequently, another inverse agonist of α5‐containing GABA_A_ receptors, https://www.guidetopharmacology.org/GRAC/LigandDisplayForward?ligandId=4299, was shown to correct cognitive deficits of Ts65Dn mice and to restore LTP in hippocampal slices after long‐term treatment (Martinez‐Cue et al., 2013). The same study showed that RO4938581 improved density of GABAergic synaptic markers in the molecular layer of the hippocampus of Ts65Dn mice. The GABA_A_ receptors containing the α5 subunit are involved in cognitive processes of learning and memory and are preferentially expressed in the hippocampus (Prut et al., 2010; Wisden, Laurie, Monyer, & Seeburg, 1992).

Although NAMs of α5‐containing GABA_A_ receptors reversed LTP deficits on hippocampal slices from Ts65Dn mice, the effects of these NAMS in vivo remained to be shown. To test the effects of a GABA_A_ α5 NAM on LTP in vivo, we first demonstrated hippocampal LTP loss in freely moving adult Ts65Dn mice. We have further shown that a single injection of α5IA to Ts65Dn mice induced a long‐lasting (at least 6 days) recovery of hippocampal LTP in vivo. We then asked whether α5IA could produce in parallel a long‐lasting effect on cognition and found that indeed a single dose of α5IA was able to reverse learning and memory deficits up to 7 days after injection. We propose that a single dose of α5IA is sufficient to induce a profound and long‐lasting remodelling of neuronal circuits involved in the cognitive functions that are affected in DS.

## 2 METHODS

### 2.1 Mouse lines

All animal care and experimental procedures complied with European Union Council (2010/63/EU), the French Ministry of Agriculture (87 848), and Spanish (BOE 252/34367‐91, 2005) guidelines for the use of laboratory animals in chronic electrophysiological and behavioural studies. In addition, French National Ethics Committees approved all experimental protocols under the accreditation numbers APAFIS3152 and 4670. Marie‐Claude Potier and Yann Hérault received official authorization from the French Ministry of Agriculture to carry out research and experiments on animals (authorization numbers 75‐2138 and 67‐369, respectively). Consistent with these guidelines, statistical analyses were used to minimize the number of animals used, within the constraints of necessary power. Animal studies are reported in compliance with the ARRIVE guidelines (Kilkenny, Browne, et al., 2010; McGrath & Lilley, 2015) and with the recommendations made by the British Journal of Pharmacology.

Ts65Dn (Ts(17^16^)65Dn) mice (MGI:4365945) were purchased from Charles River Laboratories (RRID:SCR_003792) and were bred with (C57BL/6JEiJ x C3Sn.BLiA‐Pde6b^+^/DnJ)F1 mice (MGI:2164743) also purchased from Charles River Laboratories (RRID:SCR_003792; Hoelter et al., 2008). Experiments were carried out with male Ts65Dn mice and their control littermates, during the light cycle. Studies were designed to generate, as far as possible, groups of mice of similar age and equal size with *n* ≥ 5. Four cohorts of Ts65Dn mice and their littermates were used: one cohort for in vivo electrophysiology (5 wild‐type vehicle, 5 wild‐type α5IA, 4 Ts65Dn vehicle, and 5 Ts65Dn α5IA mice), a second cohort for the novel object recognition (NOR) and the Y maze (14 wild‐type vehicle, 14 wild‐type α5IA, 13 Ts65Dn vehicle, and 13 Ts65Dn α5IA), and two additional cohorts for the Morris water maze (MWM) test (one cohort for vehicle‐treated mice and one cohort for α5IA‐treated animals; in total, 10 wild‐type vehicle, 8 wild‐type α5IA, 9 Ts65Dn vehicle, and 6 Ts65Dn α5IA mice). The number of mice for each experiment to reach statistical power was chosen based on previous studies using similar protocols in mice.

### 2.2 Genotyping

Genomic DNA was isolated from tail biopsies using the NaCl precipitation technique. Ts65Dn were identified with fast PCR protocol as described previously (Duchon et al., 2011).

### 2.3 Treatment and testing schedules

**Figure 1:**
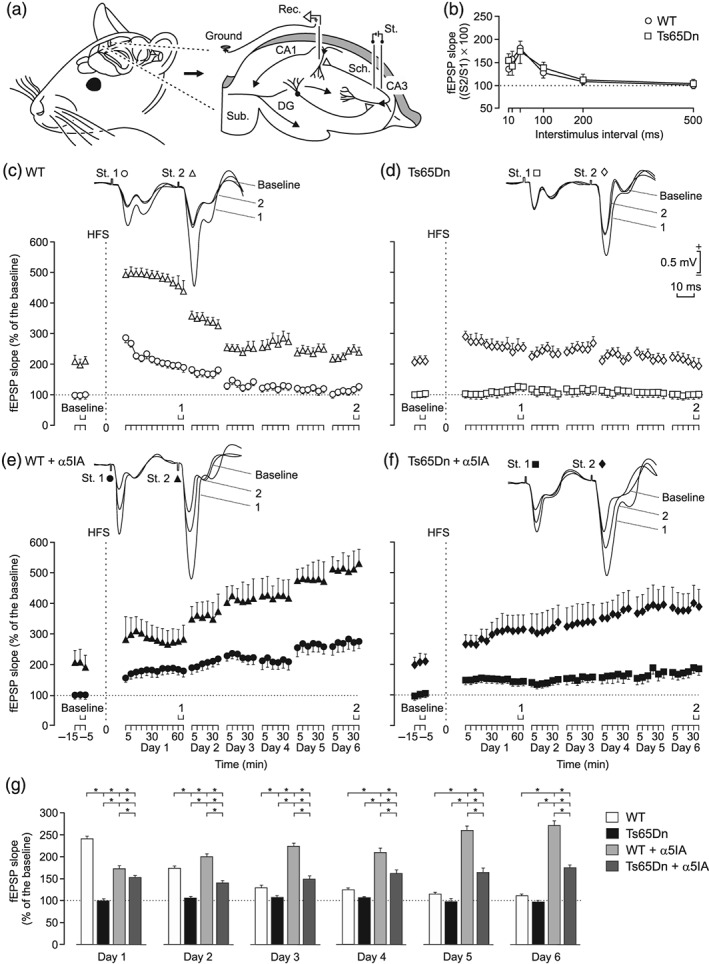
In vivo LTP deficits found at the CA3‐CA1 synapses of the hippocampus in Ts65Dn mice are sustainably reversed by a single injection of α5IA, a NAM of the α5‐containing GABA_A_ receptors. (a) Schematic diagram of the experimental design. Mice were chronically implanted with stimulating (St.) electrodes on Schaffer collaterals (Sch.) and with a recording (Rec.) electrode in the ipsilateral CA1 area of the hippocampus. An extra electrode was attached to the bone as ground. (b) Paired‐pulse facilitation evoked in WT and Ts65Dn mice at increasing time intervals. Note that peak facilitation took place at an interval of 40 ms. No significant differences were observed between the two groups of mice. (c, d) Upper traces are representative examples of field EPSPs (fEPSPs; averaged five times) collected from selected (c) WT and (d) Ts65Dn mice evoked by paired pulses (40 ms of interpulse intervals) presented to the CA3‐CA1 synapse. fEPSPs were collected before (baseline) and after the high frequency stimulation (HFS) of Schaffer collaterals at the indicated times (see 1 and 2 in the *X* axis). Lower graphs illustrate the time course of LTP evoked in the CA1 area (fEPSP mean ± *SEM*) by the paired pulses presented to Shaffer collaterals following HFS for (c) WT and (d) Ts65Dn mice. The HFS was presented after 15 min of baseline recordings, at the time marked by the dashed line. fEPSPs are given as the percentage of the baseline (100%) slope. Effects of HFS on fEPSPs evoked by the first (white circles in panel c and white squares in panel d) and second (white triangles in panel b and white diamonds in panel c) pulses are illustrated. Note that only WT mice presented a significant increase in fEPSP slopes following HFS when compared with baseline records. In addition, values collected from the WT group were significantly larger than those collected from Ts65Dn mice. (d, e) Data collected from (e) WT and (f) Ts65Dn mice following α5IA administration (5 mg· kg^−1^, i.p.). The effects of HFS on fEPSPs evoked by the first (black circles in panel e and black squares in panel f) and second (black triangles in panel e and black diamonds in panel f) pulses are illustrated. Note that α5IA facilitates the induction of LTP in control and Ts65Dn mice and that LTP was sustained in both groups of mice for at least 6 days. (g) Comparative analysis of fEPSP slopes (mean ± *SEM*) evoked by the first pulses in the four experimental groups. ^*^ *P* < .05, significantly different as indicated; ANOVA, two‐tailed. DG, dentate gyrus; Sub, subiculum

To determine the in vivo effects of α5IA, an inverse agonist of α5‐containing GABA_A_ receptors. α5IA was administered (i.p.) at a dose of 5 mg·kg^−1^, dissolved in a DMSO/Cremophor El/water (10:15:75) solution as described earlier (Braudeau, Delatour, et al., 2011). This dose was shown to be the minimal dose effective as a cognitive enhancer in WT mice as shown in figure 1b in Braudeau, Delatour, et al. (2011). For the in vivo electrophysiology, α5IA was injected 30 min before the high frequency stimulation (HFS) sessions. To assess the long‐term behavioural effects of α5IA, exposure to the NOR and Y maze tests was started 5 and 6 days after α5IA injection, respectively. For the MWM test, mice received a single dose of α5IA, 30 min prior to the first training session. Treatments were randomized, and operator and data analyses were blinded.

### 2.4 Electrophysiology

Animals were 2 to 4 months old upon their arrival at the Animal House of the Pablo de Olavide University (Seville, Spain) and were kept in collective cages (eight animals per cage) on a 12‐hr light/dark phase with constant temperature (21 ± 1°C) and humidity (50 ± 7%) until surgery. After surgery, they were maintained in individual cages. Animals were allowed ad libitum access to commercial mouse chow and water. All experiments were carried out during the light cycle.

Animals were anaesthetized with 0.8–3% halothane delivered from a calibrated Fluotec 5 (Fluotec‐Ohmeda, Tewksbury, MA, USA) vaporizer at a flow rate of 1–2 L·min^−1^ oxygen and implanted with bipolar stimulating electrodes aimed at the right Schaffer collateral‐commissural pathway of the dorsal hippocampus (2 mm lateral and 1.5 mm posterior to bregma; depth from brain surface, 1.0–1.5 mm; Paxinos & Franklin, 2001) and with a recording electrode aimed at the ipsilateral stratum radiatum underneath the CA1 area (1.2 mm lateral and 2.2 mm posterior to bregma; depth from brain surface, 1.0–1.5 mm; Figure 1a). Electrodes were made of 50 μm, Teflon‐coated tungsten wire (Advent Research Materials Ltd., Eynsham, England). The final position of hippocampal electrodes was determined using as a guide the field potential depth profile evoked by paired pulses (40 ms of interpulse interval) presented to Schaffer collaterals. A bare silver wire (0.1 mm) was affixed to the skull as ground. The four wires were connected to a four‐pin socket, and the latter was fixed to the skull with the help of two small screws and dental cement as described previously (Gruart, Muñoz, & Delgado‐Garcia, 2006).

Electrophysiological experiments started 1 week after surgery. Animals were placed in a small (5 × 5 × 5 cm) box and connected with a light wire to the recording and stimulating devices. Field EPSPs (fEPSPs) were recorded with Grass P511 differential amplifiers through a high‐impedance probe (2 × 10^12^ Ω, 10 pF). Electrical stimulus presented to Schaffer collaterals consisted of 100 μs, square, biphasic pulses applied alone, paired, or in trains.

In a preliminary experimental session aimed to characterize paired‐pulse facilitation at the CA3‐CA1 synapse, we used the pulses indicated above but presented in pairs at increasing interpulse intervals (10, 20, 40, 100, 200, and 500 ms). The stimulus intensity was set at 30–40% of the intensity necessary for evoking a maximum fEPSP response. Intervals between pairs of pulses were set at ∼30 s, to avoid unwanted interactions evoked by presynaptic or postsynaptic mechanisms (Figure 1b). Paired‐pulse ratio is defined as (fEPSP evoked by the second pulse / fEPSP evoked by the first pulse) × 100 (Gruart et al., 2006; Madroñal, Delgado‐Garcia, & Gruart, 2007).

For LTP induction, the stimulus intensity was set at 35% (0.05–0.1 mA) of values needed to evoke maximum fEPSPs. An additional criterion for selecting stimulus intensity for LTP induction was that a second stimulus, presented 40 ms after a conditioning pulse, evoked a larger (>20%) synaptic field potential than the first (Bliss & Gardner‐Medwin, 1973). After 15 min of baseline recording (one stimulus every 20 s), each animal was presented with an HFS protocol consisting of five trains (200 Hz, 100 ms) of pulses at a rate of one every second. This protocol was presented six times in total, at intervals of 1 min. Evolution of fEPSPs after the HFS protocol was followed for 60 min at the same stimulation rate (one stimulus every 20 s). Additional recording sessions (30 min) were carried out for five consecutive days.

### 2.5 Novel object recognition (NOR)

**Figure 2:**
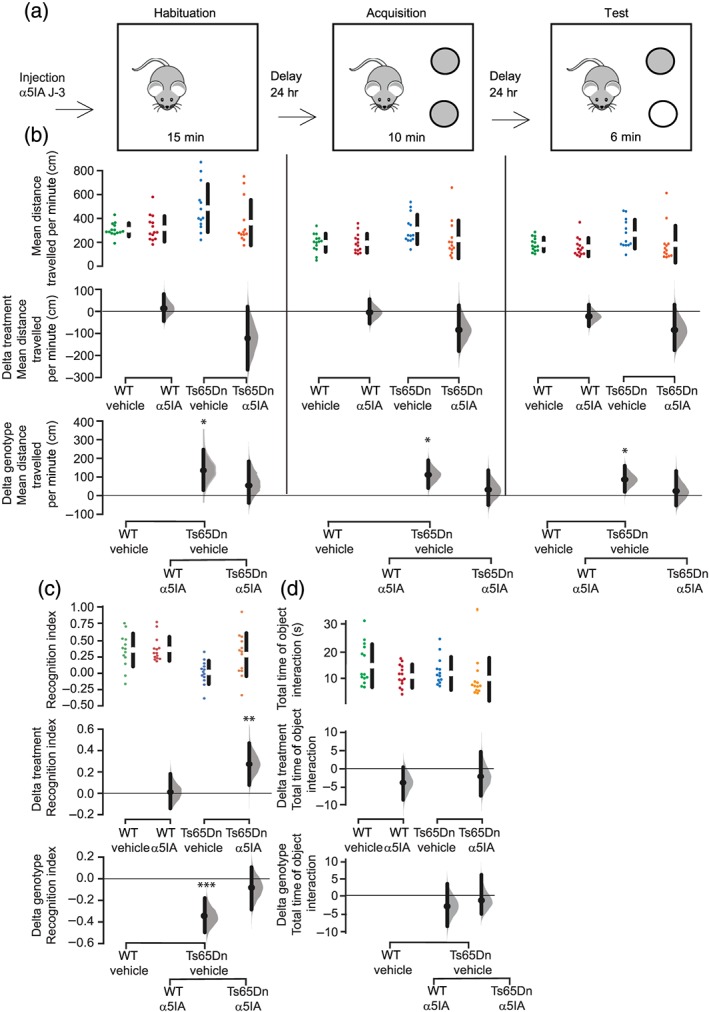
A single injection of α5IA produces a long‐term restoration of recognition memory deficits in Ts65Dn mice in the novel object recognition (NOR) task. (a) Schematic diagram of the NOR test. (b) Mean distance travelled per minute during the three sessions of the NOR (habituation, acquisition, and test). Results are shown as Cumming estimation plots. Raw data are plotted in the upper panels. Mean differences are plotted on the middle panel (treatment) and the lower panel (genotype). We calculated the 95% confidence interval of the mean difference (ends of the vertical error bars) by performing bootstrap resampling. Ts65Dn mice were hyperactive during all sessions as shown by the increase of the mean distance travelled per minute, and α5IA significantly decreased this hyperactivity during the test session. (c) Recognition index (time spent exploring the novel object over the total time spent exploring both familiar and novel objects) during the test session and statistical analyses. α5IA restored memory defects of Ts65Dn mice 5 days after treatment. ^*^ *P* < .05, significantly different as indicated

The NOR task is based on the innate tendency of rodents to differentially explore novel objects over familiar ones. On the first day of the trial, mice were habituated for 15 min to the empty circular arena (53 cm diameter) placed in a dimly lit testing room (40 lux). On the acquisition day (24 hr later), mice were placed in the arena containing two identical objects and allowed to explore them for 10 min. Mice then returned to their home cage for a 24‐hr retention interval. To test recognition memory, one familiar object and one novel object were placed in the arena, and mice were left exploring for 6 min (Figure 2a). Object exploration was scored manually and defined as the orientation of the nose to the object at a distance <1 cm. Between each trial, the arena and objects were cleaned with 70° ethanol to reduce olfactory cues. To avoid a preference for specific objects, choice of new versus familiar objects was different for the various animal groups and randomized between genotype and treatment. The location of the novel object compared to the familiar one (left or right) was randomized as well. During all these sessions, mice were video tracked (RRID:SCR_000441), and distance moved, velocity, and time spent on peripheral area were recorded.

### 2.6 Y maze

**Figure 3:**
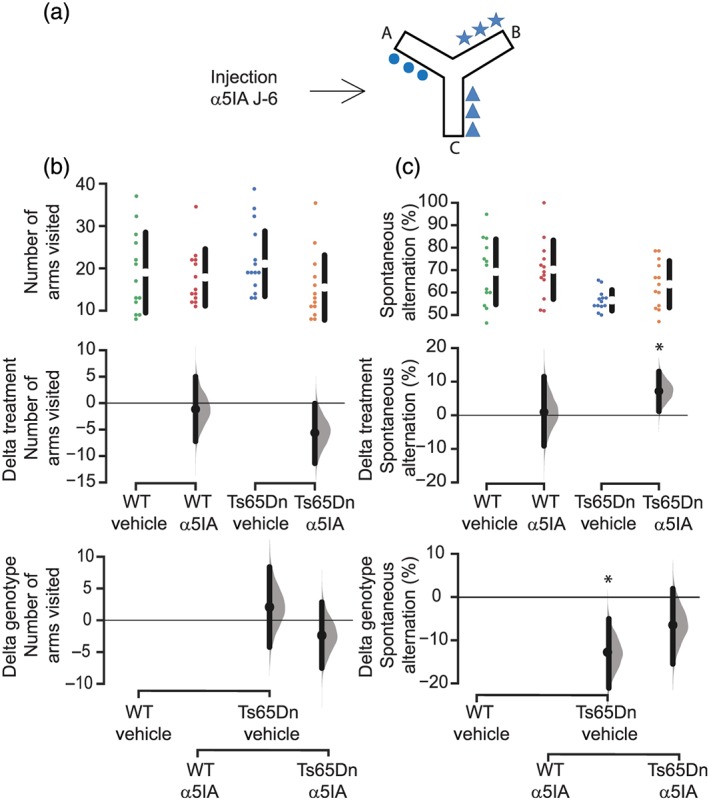
A single injection of α5IA produces a long‐term restoration of spatial working memory deficits in Ts65Dn mice in the Y‐maze task. (a) Schematic diagram of the Y‐maze test with the three types of visual cues in the three arms. (b) Number of arms visited in the Y‐maze session. Results are shown as Cumming estimation plots. Raw data are plotted on the upper panels. Mean differences are plotted on the middle panel (treatment) and the lower panel (genotype). We calculated the 95% confidence interval of the mean difference (ends of the vertical error bars). (c) Spontaneous alternation in the Y maze. Results are shown as mean ± *SEM*. We calculated the 95% confidence interval of the mean difference by performing bootstrap resampling. Working memory deficits in Ts65Dn mice were reversed 6 days after a single injection of α5IA. ^*^ *P* < .05, significantly different as indicated

Spatial working memory was assessed by recording spontaneous alternation in the Y‐maze test (Figure 3a). The Y‐maze test is based on the innate preference of animals to explore an arm that has not been previously explored, a behaviour that, if occurring with a frequency greater than 50%, is called spontaneous alternation. The basic procedure involved allowing mice free access to the three arms of the maze containing different visual cues for 8 min during which entries in the arms (whole body, including tail) were recorded. The series of arm entries were scored and alternation defined as successive entries into the three arms. The percentage of alternation was calculated as the ratio of actual to possible alternations (defined as the total number of arm entries − 2) × 100. Arm entries were scored and alternation defined as successive entries into the three arms.

### 2.7 Morris water maze (MWM)

Experiments were performed in a 150‐cm‐diameter MWM filled with opacified water kept at 20 ± 1°C and equipped with a 9‐cm‐diameter platform submerged 1 cm under the water. Mice were trained during 7 days in the standard MWM task. Training consisted in daily sessions (four trials per session). Start positions varied pseudo‐randomly among the four cardinal points. Mean intertrial interval was 30 min. During the habituation and spatial training phases, each trial ended when the animal reached the platform. A 90‐s cut‐off was used, after which mice were manually guided to the platform. Once on the platform, animals were given a 20‐s rest before being replaced in their cage. Twenty‐four hours after the last training, retention was assessed during a probe trial in which the platform was no longer available. For this probe test, all mice were released in the opposite quadrant to the target quadrant. Animals were monitored with the Any‐Maze (Stoelting, Wood Dale, USA) or the Video Track (Viewpoint, Lyon, France) video‐tracking analysis systems.

### 2.8 Data and statistical analyses

**Figure 4:**
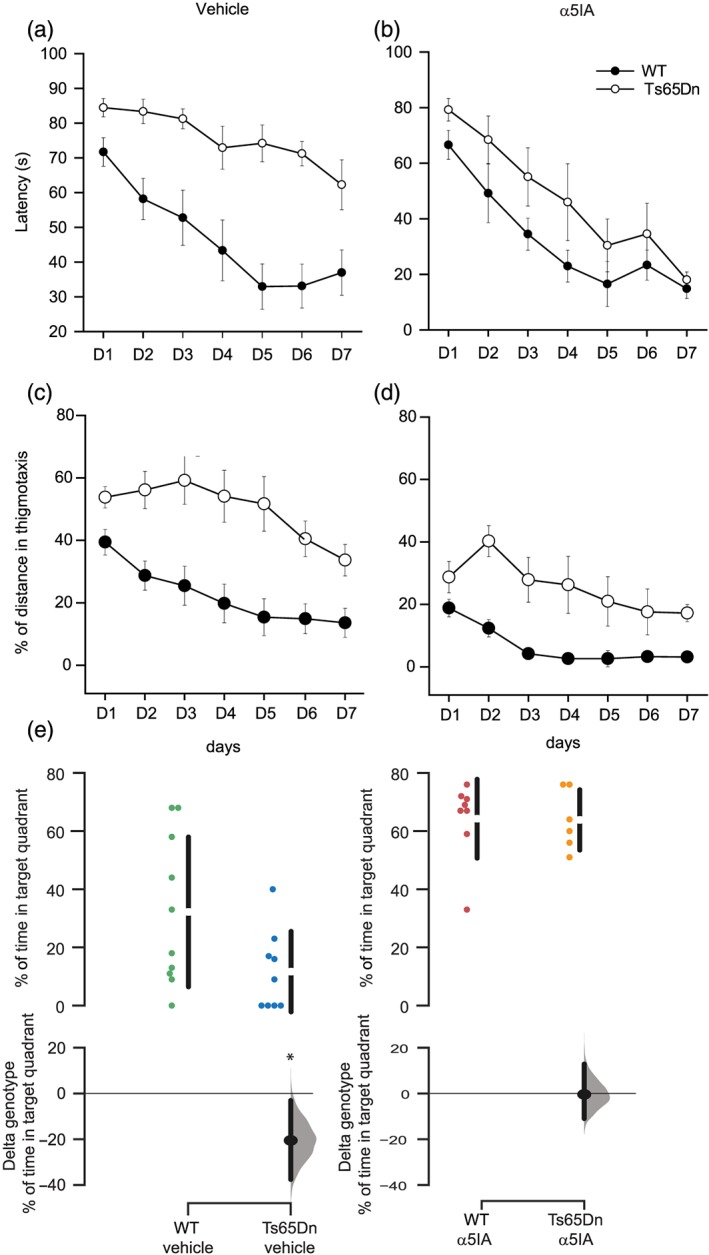
A single injection of α5IA produces a long‐term restoration of spatial memory deficits in Ts65Dn mice in the Morris water maze task. (a) Latency to reach the platform was significantly increased in vehicle‐treated Ts65Dn mice, compared with vehicle‐treated wild‐type mice. (b) On the contrary, there was no difference in latency to reach the platform in α5IA‐treated Ts65Dn mice compared to α5IA‐treated wild‐type mice (ANOVA with repeated measures). (c, d) Percentage of thigmotaxis (time spent in the 10‐cm‐wide peripheral annulus of the pool) was much higher in vehicle‐treated Ts65Dn mice, compared with vehicle‐treated wild‐type mice (c). This difference was attenuated after one injection of α5IA 30 min before the first training session but remained different between WT and Ts65Dn mice. (e) Probe test session confirmed that vehicle‐treated Ts65Dn mice spent less than 25% time in the target quadrant (e) while the other groups spent more than 25% of their exploration time in the target quadrant (e). Results are shown as mean ± *SEM*. One‐way ANOVA and ANOVA for repeated measures with Dunnett's post hoc test (a–d); 95% confidence interval of the mean difference obtained after bootstrap resampling (e)

The data and statistical analysis comply with the recommendations on experimental design and analysis in pharmacology (Curtis et al., 2018). Experiments were carried out using a double‐blind approach—namely, persons in charge of the recording and behaviour sessions and of the quantitative analyses did not have knowledge of the treatments of the experimental groups. In addition, wild‐type and Ts65Dn mice were assigned, at random, to vehicle‐ or α5IA‐injected groups. Results are shown as mean ± *SEM.* For each data set, testing for normal distribution and homogeneity of variance was performed and shown in Table S1. Statistical analysis was carried out using independent values corresponding to data obtained from different mice. For the behavioural experiments (Figures 2b,c, 3b,c, and 4e), we calculated the 95% confidence interval of the mean difference for genotype and treatment by performing bootstrap resampling (Efron, 2011). Results are presented as Cumming estimation plots. For the MWM learning phase (Figure 4a–d) and LTP (Figure 1c–g) experiments, statistical significance of differences between groups was inferred by one‐way ANOVA and ANOVA for repeated measures (data by groups), with a contrast analysis (Dunnett's post‐test if *P* value reached the threshold of .05), as there was no significant variance inhomogeneity (see Table S1). Normalization to baseline was applied to electrophysiological data to be able to compare responses before and after HFS in the same animal. No animal was excluded from the analysis of electrophysiological experiments as all of them presented data <3 SDs. Only for the MWM, one Ts65Dn mouse under vehicle was excluded as it spent more than 90 s without finding the platform for seven consecutive days and exhibited strong thigmotaxis (70% time).

A *P* value <.05 was used to define statistically significant differences between mean values. Four groups of Ts65Dn mice and their littermates were used: one for in vivo electrophysiology (for paired‐pulse facilitation: 8 wild type and 8 Ts65Dn; one for LTP: 5 wild‐type vehicle, 5 wild‐type α5IA, 5 Ts65Dn vehicle, and 5 Ts65Dn α5IA), one for the Y maze and the NOR test (14 wild‐type vehicle, 14 wild‐type α5IA, 13 Ts65Dn vehicle, and 13 Ts65Dn α5IA), and two (one for vehicle‐treated mice and one for α5IA‐treated mice) for the MWM test (10 wild‐type vehicle, 8 wild‐type α5IA, 9 Ts65Dn vehicle, and 6 Ts65Dn α5IA). All statistical data are in Table S1.

### 2.9 Materials

The drug used 3‐(5‐methylisoxazol‐3‐yl)‐6‐[(1‐methyl‐1,2,3‐triazol‐4‐yl)methyloxy]‐1,2,4‐triazolo[3,4‐a]phthalazine(a5IA)was synthesized by Orga‐Link SARL (Magny‐les‐Hameaux, France).

### 2.10 Nomenclature of targets and ligands

Key protein targets and ligands in this article are hyperlinked to corresponding entries in http://www.guidetopharmacology.org, the common portal for data from the IUPHAR/BPS Guide to PHARMACOLOGY (Harding et al., 2018), and are permanently archived in the Concise Guide to PHARMACOLOGY 2019/20 (Alexander *et al*., 2019).

## 3 RESULTS

### 3.1 LTP cannot not be evoked in vivo in Ts65Dn mice at the hippocampal CA3‐CA1 synapse

In a preliminary experimental session, we determined the paired‐pulse facilitation at the CA3‐CA1 synapse in wild‐type and Ts65Dn mice. It is generally accepted that modifications in the paired‐pulse ratio are due to changes in Ca^2+^ fluxes at the presynaptic level and that changes in synaptic strength evoked by a pair of pulses are a form of presynaptic short‐term plasticity, mostly related to variations in neurotransmitter release (Zucker & Regehr, 2002). In this regard, it has been shown that these synaptic properties can be studied in alert behaving mice (Madroñal, Delgado‐García et al., 2007). The aim was to determine the time interval (10, 20, 40, 100, 200, or 500 ms) at which both groups of animals presented the maximum facilitation, to use it during the LTP test. As illustrated in Figure 1b, the interpulse interval presenting the larger facilitation for both groups of animals was 40 ms. No significant differences were observed in the paired‐pulse facilitation between the two groups of experimental mice.

In order to obtain baseline recordings for the LTP study, animals were stimulated every 20 s for 15 min with paired pulses (40 ms of interpulse interval) at Schaffer collaterals (Figure 1a,c–f). After stable baselines were obtained, wild‐type and Ts65Dn mice were presented with the HFS protocol (see Section 2). After HFS, the same pair of pulses used to generate baseline recordings was presented at the initial rate (three every minute) for another 60 min. Recording sessions were repeated during five additional days for 30 min each (Figure 1c,d). Using this HFS and recording protocols, wild‐type mice showed a significant LTP for the first 48 hr for both the first and second pulses (Figure 1c). In contrast, Ts65Dn mice did not present any significant increase for both the first and second pulses following the HFS session (Figure 1d). Indeed, fEPSP slopes collected from wild‐type mice the first 2 days after the HFS session presented larger values than those collected from Ts65Dn mice (Figure 1c,d,g). Taken together, these results demonstrate that Ts65Dn mice did not produce LTP at the hippocampal CA3‐CA1 synapse in vivo.

### 3.2 In vivo LTP deficits in Ts65Dn mice are restored following a single injection of a selective NAM of α5‐containing GABA_A_ receptors

We previously showed that a single injection of the selective NAM of α5‐containing GABA_A_ receptors, α5IA, improved cognitive and memory deficits in the NOR task up to a level obtained in treated wild‐type mice, themselves performing better than the vehicle‐treated wild‐type mice as shown in figure 5a in Braudeau, Delatour, et al. (2011). We tested whether a single injection of α5IA would also reverse in vivo LTP deficits in Ts65Dn mice. Wild‐type and Ts65Dn mice were injected with α5IA (5 mg·kg^−1^, i.p.), 30 min before applying the HFS protocol. LTP evoked in wild‐type mice reached larger fEPSP slope values than those evoked in Ts65Dn mice for all recording sessions. Nevertheless, treatment with a single dose of α5IA induced a significant LTP in Ts65Dn for six consecutive recording days (Figure 1f,g). Noticeably, administration of α5IA to wild‐type littermates facilitated the induction of a significant delayed and long‐lasting LTP as compared with values collected in vehicle‐treated wild‐type mice (Figure 1c,e).

As already reported (Gruart et al., 2006; Madroñal et al., 2007), the paired pulses used here evoked a noticeable facilitation in the slope of the second fEPSP in relation with the first (see fEPSP traces in Figure 1c–f). Although HFS presentation modified the basal paired‐pulse ratio (see Section 2 and Figure 1b–f), no significant differences were observed between the four groups, indicating that effects evoked by α5IA did not have any presynaptic component.

Daily comparison of LTP values collected for the four groups of mice is illustrated in Figure 1g. The results further support the potentiating effect of α5IA in wild‐type mice and the rescue of LTP in Ts65Dn animals.

In summary, these results show that Ts65Dn mice did not present any noticeable synaptic potentiation following HFS of Schaffer collaterals (Figure 1d), but this situation was reversed after a single injection of a selective NAM of α5‐containing GABA_A_ receptors (Figure 1f,g). Finally, this effect was stable for at least six consecutive recording days (Figure 1f,g).

### 3.3 A single injection of a selective NAM of α5‐containing GABA_A_ receptors sustainably restores spatial working and long‐term recognition and spatial memory deficits of Ts65Dn mice

We then asked whether α5IA could induce a long‐lasting improvement of behavioural deficits in Ts65Dn mice. In order to assess hippocampal and para‐hippocampal deficits, we tested mice in the NOR paradigm, 5 days after the administration of α5IA (Figure 2a). Recognition index was calculated as the time spent investigating the novel object minus the time spent investigating the familiar object relative to the total object investigation time. Ts65Dn mice showed hyperactivity exemplified here by a higher distance travelled. α5IA treatment significantly reduced this hyperactivity especially in the test phase (Figure 2b). During the acquisition session, there was no difference of exploration time between groups of mice (data not shown). During the test session, 24 hr after the acquisition session, when one of the two familiar objects was replaced by a new one, vehicle‐treated wild‐type mice explored preferentially the novel object, compared with the familiar one, indicating that they could remember the familiar object (Figure 2c). On the contrary, Ts65Dn mice receiving vehicle were deficient as they explored both objects at the same rate (Figure 2c). Interestingly, Ts65Dn mice treated with α5IA 5 days before the test session (Figure 2a) spent more time exploring the novel object, compared with the familiar object, demonstrating that a single injection of α5IA was able to restore long‐term recognition memory deficits present in the Ts65Dn mice up to 5 days after injection (Figure 2c).

To assess spatial working memory, mice were placed in a Y maze and allowed to freely explore the three arms for 8 min (Figure 3a). The sequence of arms visited determined the percentage of alternations, calculated as the number of alternation opportunities divided by the total arm entries minus two. In order to evaluate the long‐term effects of α5IA, mice were tested 6 days after injection (Figure 3a). All groups visited arms with the same rate (Figure 3b). The percentage of spontaneous alternations was significantly lower among Ts65Dn mice compared to wild‐type littermates (Figure 3c). α5IA treatment increased the level of spontaneous alternation of Ts65Dn mice to the level of vehicle‐ or α5IA‐treated wild‐type mice. Altogether, these data show that α5IA treatment restored the spontaneous alternation deficits of Ts65Dn mice.

Finally, another group of mice was tested in the MWM for the long‐lasting effect of α5IA on long‐term spatial memory. Mice were injected with either vehicle or α5IA, 30 min before the first session and then trained without any additional treatment during the seven following days. Figure 4a shows that the latency to reach the platform was higher in Ts65Dn mice, compared with wild type receiving vehicle, while there were no significant differences between α5IA‐treated Ts65Dn and wild‐type mice (Figure 4b). Similar results were obtained when analysing distance travelled (Figure S1A,B). Mean speed remained unchanged across different conditions (Figure S1C,D), whereas thigmotaxis, corresponding to “wall seeking behaviour”, was higher in Ts65Dn mice and was reduced after α5IA treatment (Figure 4c,d).

Twenty‐four hours after the last training trial, retention was assessed during a probe trial in which the platform was no longer available. This retention test revealed that only Ts65Dn mice were not able to remember the position of the platform in the target quadrant. All three other groups (wild‐type mice receiving vehicle or α5IA and α5IA‐treated Ts65Dn mice) spent more than 25% of their time in the target quadrant. In total, one single injection of α5IA restored spatial learning proficiency and alleviated long‐term memory deficits of Ts65Dn mice up to 7 days after treatment.

## 4 DISCUSSION

The aim of our study was to assess the long‐lasting effects of α5IA, a NAM of α5‐containing GABA_A_ receptors, in a mouse model of DS. Alterations of long‐term synaptic plasticity on hippocampal brain slices from DS mouse models are mediated through GABA_A_ receptors (Kleschevnikov et al., 2012). Indeed, HFS‐induced LTP is restored after incubating hippocampal slices from Ts65Dn mice with GABA_A_ receptor antagonists (Kleschevnikov et al., 2012). In addition, chronic treatment of Ts65Dn mice with either the non‐selective GABA_A_ antagonist pentylenetetrazol or an NAM of the α5‐containing GABA_A_ receptors, RO4938581, with a chemical structure different from α5IA, restored LTP induction on hippocampal slices (Fernandez et al., 2007; Martinez‐Cue et al., 2013). Knocking down one copy of the *Gabra5* gene in the Ts65Dn mice led to a robust enhancement of LTP in hippocampal slices (Vidal et al., 2018). Finally, differentially expressed proteins involved in molecular pathways defining LTP show abnormal profiles in vehicle‐treated Ts65Dn as compared to WT mice and a rescue following chronic treatment with RO4938581 (Block et al., 2018).

We investigated the effects of an NAM of α5‐containing GABA_A_ receptors, α5IA, on LTP using electrophysiological recordings in vivo. We analysed for the first time hippocampal LTP in Ts65Dn mice in vivo. We found that LTP was not evoked at the hippocampal CA3‐CA1 synapse in freely moving Ts65Dn mice following HFS and that a single injection of α5IA to chronically implanted Ts65Dn mice could induce a long‐lasting (at least 6 days) recovery of hippocampal LTP loss in vivo. These results are in agreement with our previous study showing that α5IA increased expression of IEGs associated with hippocampal LTP in vivo (French et al., 2001), during memory processing in wild‐type mice and normalized the lower level of IEG expression in Ts65Dn mice (Braudeau, Dauphinot, et al., 2011).

Apart from restoring the susceptibility to LTP induction in Ts65Dn mice, the injection of α5IA also changed the time course of potentiation during the days following HFS in both wild‐type and Ts65Dn littermates. Although, in most reported situations, LTP decreases with time with rather different time constants (Abraham & Williams, 2003; Madroñal et al., 2007), there are reports describing the presence of LTP that increases across time (Jurado‐Parras, Gruart, & Delgado‐Garcia, 2012). On the other hand, it has been already reported that learning‐dependent potentiation of synaptic strength increases across training sessions (Gruart et al., 2006). In this context, it is possible that α5IA administration activated some of the molecular processes involved in the acquisition of actual learning, thus leading to an increased susceptibility of synapses to undergo potentiation.

Distal dendritic inhibition of pyramidal neurons in the primary somatosensory cortex is mediated by α5‐containing GABA_A_ receptors (Ali & Thomson, 2008). A recent study showed that suppression of dendritic inhibition in the motor cortex leads to increased spine formation during a motor learning task (Chen, Kim, Peters, & Komiyama, 2015). In addition, the α5 subunit is more concentrated in the dendrites of hippocampal CA1 pyramidal cells (Fritschy & Mohler, 1995) and mediates tonic and synaptic inhibition (Schulz, Knoflach, Hernandez, & Bischofberger, 2018). Furthermore, increased α5‐mediated dendritic, but not somatic, inhibition impaired LTP induction in Ts65Dn mice (Schulz, Knoflach, Hernandez, & Bischofberger, 2019). It is thus possible that reduced dendritic inhibition caused by α5IA injections allows the formation of new synaptic contacts that restore the susceptibility of the circuit to undergo plastic changes during, at least, several days.

As LTP evaluates synaptic strength and plasticity and has been proposed as a molecular substrate of learning and memory (Bliss & Gardner‐Medwin, 1973), we hypothesized, based on the long‐term recovery of LTP in vivo in Ts65Dn mice following a single injection of α5IA, that α5IA could also induce long‐term recovery of memory deficits in Ts65Dn mice. Impressively, α5IA reversed learning and memory deficits in Ts65Dn mice, up to 7 days after injection in several behavioural tasks assessing hippocampal‐ and cortical‐dependent spatial/nonspatial, working/reference and long‐term memory deficits, suggesting that α5IA produces long‐lasting pharmacological effects by sustainably restoring in vivo LTP in Ts65Dn mice. Thus, a single injection of α5IA could profoundly modify the neuronal networks that are the cellular substrate of LTP and learning and memory in the hippocampal and cortical regions. Alternatively, the long‐term effects of α5IA could be due to pharmacokinetic properties of the drug allowing its accumulation in brain and/or plasma for more than 1 week after i.p. injection and producing unusually sustained target engagement.

The pharmacokinetic properties of α5IA have been studied extensively. Given orally to rats, α5IA was stable in plasma within 20 hr and then decreased. α5IA receptor occupancy in vivo was dose and time dependent with maximum occupancy occurring within the first 2 hr and decreasing after 20 hr (Atack et al., 2009). α5IA had moderate to high clearance rates, a short half‐life, and a good bioavailability in rats. However, although oral bioavailability was good in rats, it was poor in dog and rhesus monkey (Atack, 2010). The pharmacokinetic study published which is the closest to our experimental conditions is the one from Dawson et al. where the authors measured in vivo receptor occupancy after a single i.p. injection of α5IA at 10 mg·kg^−1^ in 70% polyethylene glycol/30% water in mice, twice the dose used in our study but using another excipient. Maximal occupancy (94%) occurred after 1 hr and was still 50% after 8 hr (Dawson et al., 2006). In our study, long‐term effects on LTP induction and memory tests were observed up to 7 days when plasma levels of α5IA and receptor occupancy are expected to be too low to elicit any of these effects.

We can thus predict that the long‐term effects of α5IA in Ts65Dn mice were not due to its pharmacokinetic properties, maintaining sufficient plasma concentrations of the drug, but rather to long‐lasting changes of synaptic plasticity. This hypothesis fits with our results on LTP recovery in vivo in Ts65Dn mice and the study that tested the effects of α5IA on LTP induction after HFS on hippocampal slices in wild‐type mice. Changes in synaptic plasticity were enhanced by α5IA. The fEPSP slope was significantly increased, compared with vehicle, with a slower decrease 2 hr after burst (Dawson et al., 2006). A persistent therapeutic effect of a novel selective antagonist of the α5‐containing GABA_A_ receptors has recently been reported in rodent models of vascular cognitive impairment, following a wash‐out period of up to 13 days (Gacsalyi et al., 2018).

Inhibitory interneurons connecting with either the soma or dendrites of pyramidal neurons could prevent the formation of plasticity as shown by the lack of LTP induction in vivo at the CA3‐CA1 synapse. In this regard, results reported here for LTP evoked by pair of pulses further suggest that changes were evoked post‐synaptically (Gruart et al., 2006; Madroñal et al., 2007).

While no change in the expression of the *Gabra5* gene nor in the amount of protein encoded by this gene has been found in Ts65Dn mice (Braudeau, Delatour, et al., 2011; Martinez‐Cue et al., 2013), there are no results published yet on the amount of α5‐containing GABA_A_ receptors in individuals with DS by PET. These receptors have been shown to be altered in human diseases such as Alzheimer's disease (increase in post mortem brains in the CA1 region and decrease in the superior temporal gyrus; Kwakowsky et al., 2018). A decrease of α5‐containing GABA_A_ receptors in autism spectrum disorder has also been demonstrated by PET (Mendez et al., 2013).

In healthy humans, pretreatment with α5IA significantly reduced the amnesic effect of alcohol, again demonstrating that α5 GABA_A_ receptors play a principal role in memory processes (Nutt, Besson, Wilson, Dawson, & Lingford‐Hughes, 2007). However, treatment with another NAM of the α5‐containing GABA_A_ receptors, RO5186582 also named RG1662 and https://www.guidetopharmacology.org/GRAC/LigandDisplayForward?ligandId=10427, given orally twice daily at two doses for 26 weeks to 173 12‐ to 30‐year‐old individuals with DS, did not show significant effects on cognition and adaptive behavior, as reported briefly by Roche, but still unpublished (CLEMATIS Phase 2 randomized double‐blind placebo‐controlled trial, NCT02024789). This led to discontinuation of another clinical trial in a paediatric population (age 6–11 years; NCT02484703). As this unpublished α5 NAM has a chemical structure (US 8,835,425 B2) different from α5IA and RO4938581, it will be important to compare their activities and mechanisms of action in cellular and mouse models of DS with α5IA and RO4938581, using the same protocols. Indeed, α5IA is a non‐selective ligand of the GABA_A_ receptors that has selective negative efficacy on the α5‐containing GABA_A_ receptors while RO4938581 binds selectively to the α5‐containing GABA_A_ receptors and has negative intrinsic efficacy (Ballard et al., 2009; Sternfeld et al., 2004). For basmisanil, no data are publicly available under basmisanil, RO5186582, or RG1662. Public access to the data from the CLEMATIS study is essential to understand the reported lack of efficacy versus a placebo effect, based on a composite score from various cognitive scales assessing language, executive functions, and memory (Liogier d'Ardhuy et al., 2015).

In conclusion, our results highlight the relevance of selective NAMs of the α5‐containing GABA_A_ receptors for treating cognitive deficits, particularly in individuals with DS, based on the long‐lasting pharmacological effects of α5IA in Ts65Dn mice. Studies on long‐term functional remodelling of specific GABAergic synapses following treatment with NAMs of the α5‐containing GABA_A_ receptors are eagerly awaited.

## CONFLICT OF INTEREST

The authors declare no conflicts of interest.

## AUTHOR CONTRIBUTIONS

M.‐C.P., Y.H., and J.M.D.‐G. designed the study. A.D., A.G., and C.A. did the experiments. A.D., A.G., J.M.D.‐G., B.D., and J.Z.S.M. analysed the data and prepared the figures. M.‐C.P., Y.H., J.M.D.‐G., A.D., B.D., and J.Z.S.M. wrote the paper. All authors contributed to the writing and revisions and approved the final version.

## DECLARATION OF TRANSPARENCY AND SCIENTIFIC RIGOUR

This Declaration acknowledges that this paper adheres to the principles for transparent reporting and scientific rigour of preclinical research as stated in the *BJP* guidelines for https://bpspubs.onlinelibrary.wiley.com/doi/full/10.1111/bph.14207, and https://bpspubs.onlinelibrary.wiley.com/doi/full/10.1111/bph.14206, and as recommended by funding agencies, publishers, and other organizations engaged with supporting research.

## Supporting information

Table S1. Statistical analyses of in vivo LTP and behavioral experiments. Distance travelled was higher in Ts65Dn mice as compared to wild‐type under vehicle (**A**) wile speed was similar (**C**) while there were no significant differences between α5IA‐treated Ts65Dn and wild‐type mice for distance travelled (**B**) and speed (**D**).

Figure S1. A single injection of α5IA restores distance travelled in Ts65Dn mice in the Morris water maze task.
