# Supplementary material for: Long‐lasting correction of in vivo LTP and cognitive deficits of mice modelling Down syndrome with an α5‐selective GABAA inverse agonist
Source: Br J Pharmacol. 2020 Jan 9;177(5):1106–18. doi: 10.1111/bph.14903 (PMC7042104; doi:10.1111/bph.14903)
Supplement: Supplementary file 1 — Table S1. Statistical analyses of in vivo LTP and behavioral experiments. Distance travelled was higher in Ts65Dn mice as compared to wild‐type under vehicle (A) wile speed was similar (C) while there were no significant differences between α5IA‐treated Ts65Dn and wild‐type mice for distance travelled (B) and speed (D). [file BPH-177-1106-s001.pdf]

Figure 1B — Double pulse

	Test per	Condition group 1	Condition group 2	All pairwise multiple comparison procedures (Holm-Sidak method): mean difference	t value	*p*	Statistical differences	Normality Test (Shapiro-Wilk)	Equal Variance Test (Brown-Forsythe)	Two way repeated measures ANOVA (One factor repetiton, Interval)
	Treatment x Interval=10 ms	wt Vehicle	Ts65Dn vehicle	17 708	0.823	0.415	No	Passed, *p* =0.06	Passed, *p* =0.816	F(5,70)=0.471, *p* =0.797
	Treatment x Interval=20 ms	wt Vehicle	Ts65Dn vehicle	13 741	0.638	0.526	No			
	Treatment x Interval=40 ms	wt Vehicle	Ts65Dn vehicle	4 969	0.231	0.818	No			
	Treatment x Interval=100 ms	wt Vehicle	Ts65Dn vehicle	11 387	0.529	0.599	No			
	Treatment x Inteval=200 ms	wt Vehicle	Ts65Dn vehicle	0.747	0.0347	0.972	No			
	Treatment x Inteval=200 ms	wt Vehicle	Ts65Dn vehicle	5 908	0.274	0.785	No			

Figure 1 C-F — LTP Test-evolution

	Test per	Condition group 1	Condition group 2		Normality Test (Shapiro-Wilk)	Equal Variance Test (Brown-Forsythe)	Two way repeated measures ANOVA (One factor repetiton, Interval)
	Treatments x Time (min) from 15 min before High Frequency Stimulation (HFS) to 60 min after HFS. 30 min during the five following days were also recorded. Data are represented every 5 min.	Wt Vehicle	Wt+a5IA	Too many comparisions to be indicated individually. Overall, 45 measured times (from Baseline to Day 6) by 6 group comparisons.	Passed, *p* =0.08	Passed, *p* =0.115	F(114,608)=8.452, *p* <0.001
		Wt Vehicle	Ts6Dn vehicle				
		Wt Vehicle	Ts65Dn α5IA				
		Wt+a5IA	Ts6Dn vehicle				
		Wt+a5IA	Ts65Dn α5IA				
		Ts65Dn vehicle	Ts65Dn α5IA				

Figure 1 G — LTP Test- groups x days

	Test per	Condition group 1	Condition group 2	All pairwise multiple comparison procedures (Holm-Sidak method): mean difference	t value	p	Statistical differences	Normality Test (Shapiro-Wilk)	Equal Variance Test (Brown-Forsythe)	ANOVA
Day 1	Treatment	Wt Vehicle	Wt+a5IA	67.66	8 829	*p* <0.001	Yes	Passed, *p* =0.399	Passed, *p* =0.130	F(3,96)=116.026, *p* <0.001
		Wt Vehicle	Ts6Dn vehicle	140 994	18 399	*p* <0.001	Yes			
		Wt Vehicle	Ts65Dn α5IA	88 695	11 574	*p* <0.001	Yes			
		Wt+a5IA	Ts6Dn vehicle	73 334	9 570	*p* <0.001	Yes			
		Wt+a5IA	Ts65Dn α5IA	21 035	2,745	*p* =0.007	Yes			
		Ts65Dn vehicle	Ts65Dn α5IA	52 299	6 825	*p* <0.001	Yes			
Day 2	Treatment	Wt Vehicle	Wt+a5IA	27 151	4 223	*p* <0.001	Yes	Passed, *p* =0.008	Passed, *p* =0.711	F(3,96)=79.507, *p* <0.001
		Wt Vehicle	Ts6Dn vehicle	66 717	10 377	*p* <0.001	Yes			
		Wt Vehicle	Ts65Dn α5IA	31 921	4 965	*p* <0.001	Yes			
		Wt+a5IA	Ts6Dn vehicle	93 868	14.6	*p* <0.001	Yes			
		Wt+a5IA	Ts65Dn α5IA	59 072	9 188	*p* <0.001	Yes			
		Ts65Dn vehicle	Ts65Dn α5IA	34 796	5 412	*p* <0.001	Yes			
Day 3	Treatment	Wt Vehicle	Wt+a5IA	93 839	11 142	*p* <0.001	Yes	Failed, *p* <0.05	Passed, *p* =0.435	F(3, 96)=71.018, *p* <0.001
		Wt Vehicle	Ts6Dn vehicle	21 742	2 581	*p* =0.023	Yes			
		Wt Vehicle	Ts65Dn α5IA	20 218	2 401	*p* =0.018	Yes			
		Wt+a5IA	Ts6Dn vehicle	115 581	13 723	*p* <0.001	Yes			
		Wt+a5IA	Ts65Dn α5IA	73 621	8,741	*p* <0.001	Yes			
		Ts65Dn vehicle	Ts65Dn α5IA	41.96	4 982	*p* <0.001	Yes			
Day 4	Treatment	Wt Vehicle	Wt+a5IA	87 052	8 063	*p* <0.001	Yes	Failed, *p* <0.05	Passed, *p* =0.102	F(3, 96)=36.95, *p* <0.001
		Wt Vehicle	Ts6Dn vehicle	17 797	1 648	*p* =0.103	No			
		Wt Vehicle	Ts65Dn α5IA	37 918	3 512	*p*= 0.001	Yes			
		Wt+a5IA	Ts6Dn vehicle	104 849	9 711	*p* <0.001	Yes			
		Wt+a5IA	Ts65Dn α5IA	49 134	4 551	*p* <0.001	Yes			
		Ts65Dn vehicle	Ts65Dn α5IA	55 715	5.16	*p* <0.001	Yes			
Day 5	Treatment	Wt Vehicle	Wt+a5IA	145 338	15 106	*p* <0.001	Yes	Passed, *p* =0.089	Passed, *p* =0.078	F(3, 96)=93.359, *p* <0.001
		Wt Vehicle	Ts6Dn vehicle	14 544	1.37	*p* =0.174	No			
		Wt Vehicle	Ts65Dn α5IA	52-047	4 904	*p* <0.001	Yes			
		Wt+a5IA	Ts6Dn vehicle	160 338	15 106	*p* <0.001	Yes			
		Wt+a5IA	Ts65Dn α5IA	93 747	8 832	*p* <0.001	Yes			
		Ts65Dn vehicle	Ts65Dn α5IA	66 591	5 274	*p* <0.001	Yes			
Day 6	Treatment	Wt Vehicle	Wt+a5IA	159 562	15 179	*p* <0.001	Yes	Failed, p<0.05	Passed, *p* =0.114	F(3, 96)=114.507, *p* <0.001
		Wt Vehicle	Ts6Dn vehicle	15 416	1 466	*p=1.466*	No			
		Wt Vehicle	Ts65Dn α5IA	64 717	6 156	*p* <0.001	Yes			
		Wt+a5IA	Ts6Dn vehicle	174 977	16 645	*p* <0.001	Yes			
		Wt+a5IA	Ts65Dn α5IA	94 844	9 022	*p* <0.001	Yes			
		Ts65Dn vehicle	Ts65Dn α5IA	80 133	7 623	*p* <0.001	Yes			

Figure 3 — Y maze

Variable	Test per	Condition group 1	Condition group 2	mean difference	95 CI lower limit	95 CI upper limit	95 CI containing 0	Normality Test (Shapiro-Wilk)	Equal Variance Test (Brown-Forsythe)	ANOVA
Number of arms visited	Treatment	wt Vehicle	wt α5IA	-1.153	-7.384	4.692	yes	Failed, *p* < 0.050	Not Done	Not Done
		Ts65Dn vehicle	Ts65Dn α5IA	-5.615	-11.307	0.000	yes			
	genotype	wt vehicle	Ts65Dn vehicle	2.076	-4.307	8.230	yes			
		wt α5IA	Ts65Dn α5IA	-2.384	-7.615	3.076	yes			
% spontaneous alternation	Treatment	wt Vehicle	wt α5IA	0.957	-9.112	11.511	yes	Passed, *p* =0.869	Failed *p* <0.050	Not Done
		Ts65Dn vehicle	Ts65Dn α5IA	7.164	1.149	13.029	**no**			
	genotype	wt vehicle	Ts65Dn vehicle	-12.691	-20.766	-4.757	**no**			
		wt α5IA	Ts65Dn α5IA	-6.485	-15.607	1.934	yes			

Figure 2 — Novel object recognition

Variable	Test per	Condition group 1	Condition group 2	mean difference	95 CI lower limit	95 CI upper limit	95 CI containing 0	Normality Test (Shapiro-Wilk)	Equal Variance Test (Brown-Forsythe)	ANOVA
Habituation Mean distance travelled (cm)	Treatment	wt Vehicle	wt α5IA	73.072	-16.627	159.501	yes	Failed, *p* < 0.050	Not Done	Not Done
		Ts65Dn vehicle	Ts65Dn α5IA	84.237	-50.003	226.345	yes			
	genotype	wt vehicle	Ts65Dn vehicle	181.865	76.212	293.642	no			
		wt α5IA	Ts65Dn α5IA	46.287	-57.464	165.232	yes			
Aquisition Mean distance travelled (cm)	Treatment	wt Vehicle	wt α5IA	169.321	74.158	272.476	**no**	Failed, *p* < 0.050	Not Done	Not Done
		Ts65Dn vehicle	Ts65Dn α5IA	117.688	-6.699	243.119	yes			
	genotype	wt vehicle	Ts65Dn vehicle	111.592	40.841	189.277	**no**			
		wt α5IA	Ts65Dn α5IA	31.854	-50.465	135.625	yes			
Test Mean distance travelled (cm)	Treatment	wt Vehicle	wt α5IA	95.451	24.270	167.624	**no**	Failed, *p* < 0.050	Not Done	Not Done
		Ts65Dn vehicle	Ts65Dn α5IA	62.272	-31.601	152.893	yes			
	genotype	wt vehicle	Ts65Dn vehicle	86.411	20.554	160.094	**no**			
		wt α5IA	Ts65Dn α5IA	24.538	-52.939	131.935	yes			
Recognition index	Treatment	wt Vehicle	wt α5IA	-1.293	-21.331	18.353	yes	Passed, *p* =0.869	Passed, *p* =0.114	*F* (1,50)=4.548, *p* =0.038
		Ts65Dn vehicle	Ts65Dn α5IA	23.516	4.570	44.437	**no**			
	genotype	wt vehicle	Ts65Dn vehicle	-0.343	-0.496	-0.178	**no**	Passed, *p* =0.869	Passed, *p* =0.114	*F* (1,50)=10.272, *p* =0.002
		wt α5IA	Ts65Dn α5IA	-0.0823	-0.285	0.106	yes			

Figure 4 — Morris Water Maze

Variable	Test per	Condition group 1	Condition group 2	mean difference	95 CI lower limit	95 CI upper limit	95 CI containing 0	Normality Test (Shapiro-Wilk)	Equal Variance Test (Brown-Forsythe)	ANOVA
Latency (sec)	genotype	wt vehicle	Ts65Dn vehicle					Passed, *p* =0.722	Passed, *p* =0.828	RM *F* (1,102)=25.343, *p* <0.001
	genotype	wt α5IA	Ts65Dn α5IA					Passed, *p* =0.632	Passed, *p* =0.643	RM *F* (1,72)=3.035, *p* =0.107
Distance (m)	genotype	wt vehicle	Ts65Dn vehicle					Passed, *p* =0.559	Passed, *p* =0.832	RM *F* (1,102)=12.375, *p* =0.003
	genotype	wt α5IA	Ts65Dn α5IA					Passed, *p* =0.819	Passed, *p* =0.346	RM *F* (1,72)=3.970, *p* =0.07
% distance in thigmotaxis	genotype	wt vehicle	Ts65Dn vehicle					Passed, *p* =0.179	Passed, *p* =0.181	RM *F* (1,102)=17.225, *p* <0.001
Log 1+% distance in thigmot	genotype	wt α5IA	Ts65Dn α5IA					Passed, *p* =0.310	Passed, *p* =0.990	RM *F* (1,72)=25,670, *p* <0.001
Mean speed	genotype	wt vehicle	Ts65Dn vehicle					Passed, *p* =0.065	Passed, *p* =0.240	RM *F* (1,102)=1.616, *p* =0.221
Log 1-Mean speed	genotype	wt α5IA	Ts65Dn α5IA					Passed, *p* =0.298	Passed, *p* =0.720	RM *F* (1,72)=0,148, *p* =0.707
Probe test	genotype	wt vehicle	Ts65Dn vehicle	-20.533	-38.255	-3.588	**no**	Passed, *p* =0.243	Passed, *p* =0.079	*F* (1,17)=4.531, *p* =0.048
		wt α5IA	Ts65Dn α5IA	-0.416	-10.625	12.708	yes	Failed, *p* < 0.050	Passed, *p* =0.936	Not Done
