# Supplementary figures and images for: Long‐lasting correction of in vivo LTP and cognitive deficits of mice modelling Down syndrome with an α5‐selective GABAA inverse agonist
Source: Br J Pharmacol. 2020 Jan 9;177(5):1106–18. doi: 10.1111/bph.14903 (PMC7042104; doi:10.1111/bph.14903)

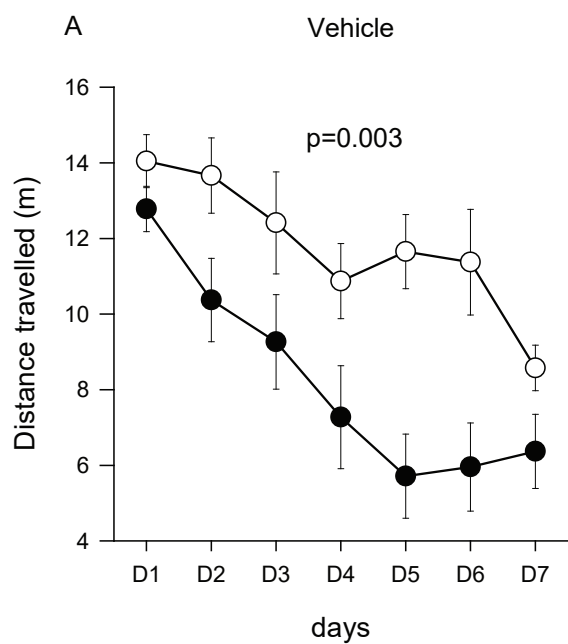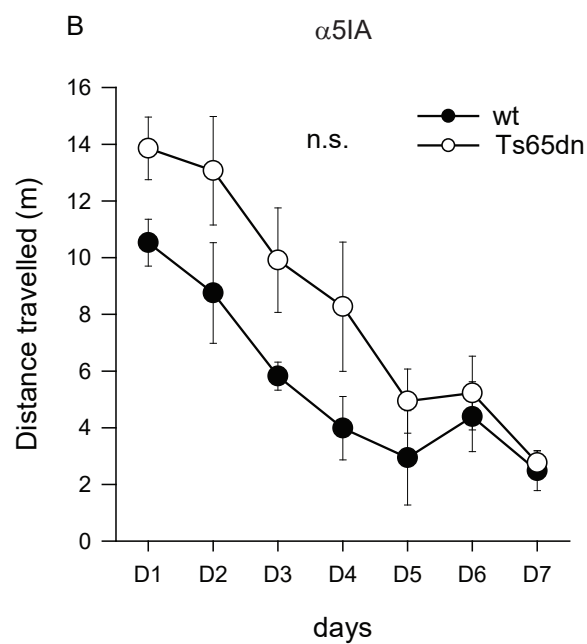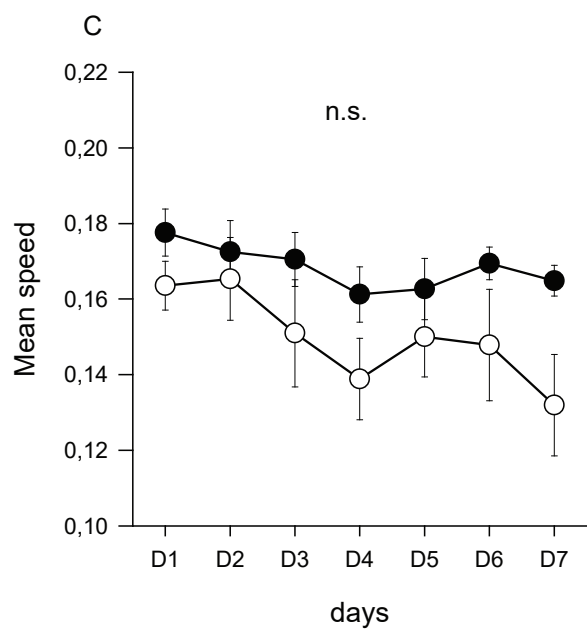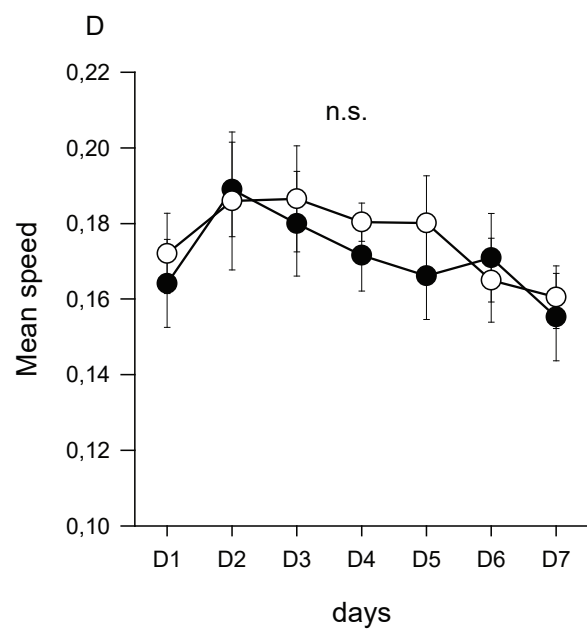

Supplement: Supplementary file 2 — Figure S1. A single injection of α5IA restores distance travelled in Ts65Dn mice in the Morris water maze task. [file BPH-177-1106-s002.pdf]
